# A Review on the Mechanism and Application of Keishibukuryogan

**DOI:** 10.3389/fnut.2021.760918

**Published:** 2021-12-24

**Authors:** Koichiro Tanaka, Koki Chiba, Kazuhiko Nara

**Affiliations:** Department of Traditional Medicine, Faculty of Medicine, Toho University, Tokyo, Japan

**Keywords:** Kampo, blood stasis, Oketsu, TCM, traditional medicine, Keishibukuryogan, gui-zhi-fu-ling-wan

## Abstract

The concept of “blood stasis” – called yū xiě in Chinese, Oketsu in Japanese – is one of the unique pathophysiology of traditional medicine that originated in China and inherited in Korea and Japan. This concept is related to the multiple aspects of hemodynamic disorders brought on by quantitative and qualitative changes. It theorizes that the quantitative changes of “blood stasis” are related to peripheral circulatory insufficiency. When chronic qualitative changes of “blood stasis” produce stagnant blood that turns into a pathological product, it could cause inflammation and lead to organic changes. Trauma induced hematomas, that are considered to be a quantitative change of blood, are also a form of blood stasis. The basic medicine research on Keishibukuryogan (KBG)–a Japanese name in Traditional Japanese Medicine (Kampo) for one of the most common anti- “blood stasis” prescriptions, also known as gui-zhi-fu-ling-wan (GFW) in Chinese in Traditional Chinese Medicine (TCM)–indicated that the initiation of quantitative changes was closely related to loss of redox balances on endothelial function induced by oxidative stress. The following qualitative changes were related to coagulopathy, hyper viscosity; anti-platelet aggregation, lipid metabolism; a regulation of systemic leptin level and/or lipid metabolism, inflammatory factor; cyclooxygenase-1,2 (COX-1, 2), interleukin-6, 8 tumor necrosis factor-α, macrophage infiltration, hyperplasia, tissue fibrosis and sclerosis caused by transforming growth factor-β1 and fibronectin, the dysfunction of regulated cell deaths, such as, apoptosis, autophagy, ferroptosis and ovarian hormone imbalance. Clinically, KBG was often used for diseases related to Obstetrics and Gynecology, Endocrine Metabolism, Rheumatology and Dermatology. In this review, we give an overview of the mechanism and its current clinical application of KBG through a summary of the basic and clinical research and discuss future perspective.

## Introduction

The concept of “blood stasis” -called yu xiě in Chinese, Oketsu in Japanese–is one of the unique pathophysiology of traditional medicine that originated in China and inherited in Korea and Japan. This concept is related to the multiple aspects of hemodynamic disorders brought on by quantitative and qualitative changes. It theorizes that the quantitative changes in the blood are related to peripheral circulatory insufficiency. According to 211 studies about “blood stasis” in Korean Traditional Medicine as well, which were 19 reviews, 52 clinical studies and 140 preclinical studies, “stagnant blood within the body” was the most frequently mentioned phrase of the traditional concept of blood stasis, followed by “disorder of blood circulation,” “pathological product,” “the blood lost its physiological function,” “extravasated blood,” “blood congested in viscera and tissue,” “foul blood,” “blood congested in a blood vessel,” “organ dysfunction” and “stagnation of blood flow in local parts.” Among these, the quantitative concepts of peripheral circulation disorders are suggested by “disorder of blood circulation,” “blood congested in viscera and tissue,” “blood congested in a blood vessel” and “stagnation of blood flow in local parts.” “Pathological product,” “the blood lost its physiological function,” “foul blood” suggested qualitative changes due to chronic “blood stasis.” In the preclinical studies in Korea, coagulopathy was studied most frequently, followed by hyper viscosity, hyperlipidemia, inflammation, neoplasm, ischemic brain injury, and atherosclerosis. In the clinical studies, traumatic injury was the most frequently studied disease/condition, followed by genitourinary and cerebrovascular disease (1). In this review, we give an overview of the mechanism and its current clinical application of KBG–a Japanese name in Traditional Japanese Medicine (Kampo) for one of the most common anti- “blood stasis” prescriptions, also known as gui-zhi-fu-ling-wan (GFW) in Chinese in Traditional Chinese Medicine (TCM)-through a summary of the basic and clinical research and discuss future perspective.

## Basic Research

The effects of KBG in basic research are mainly about antioxidant, Nitric Oxide (NO) production, vasodilatory effects, and suppression of inflammatory cytokine production, all of which are closely related. The concepts of qualitative and quantitative changes of “blood stasis” overlapped. Peripheral circulatory insufficiency, a quantitative change, is likely to cause micro-inflammation due to active oxygen and inflammatory cytokines. Chronic qualitative changes altered lipid metabolism and dynamics of female hormones and produced “stagnant blood” that turns into a pathological product, which could cause inflammation and lead to organic changes, such as hyperplasia, fibrosis and sclerosis. These results are summarized on Table 1.

**Table 1:** The effects of KBG in basic medicine research.

**Effects**	**Related substances**	**Related pathways**	**Targets**	**Reference**
vasodilation	NO, EDRF		endothelial cell	(2, 3)
anti-platelet aggregation			platelet	(4)
antioxidant	lipid oxidation, superoxide dismutase, xanthine oxidase, VCAM-1		endothelial cell, erythrocyte	(3, 5–13)
anti-inflammation	NO, MIF, IL-1β, 6, 8, PGE-2, TNF-α, COX-1,2,	TNF, NF-kappa B		(8, 9, 14, 15)
macrophage infiltration	MCP-1, VCAM-1, osteopontin			(8)
anti-fibrotic (accumulation of ECM)	TGF-β1, fibronectin, fibroblast proliferation, TNF-α, MIP-2, IL-6, collagen production,	VEGF signaling, Toll-like receptor signaling	kidney, liver	(8, 9, 12, 16, 17)
lipid metabolism	adipocytokine (leptin, TNF-alpha), adiponectin		adipocyte, liver, epididymis	(18, 19)
against atherosclerosis	lipid peroxidase, oxidative LDL modification			(10, 11)
regulated cell deaths	ferroptosis,	p62-Keap 1-NRF2 p	endometrial hyperplasia	(13)
	autophagy,	PI3K/AKT/mTOR	uterus	(20)
hot flash	CGRP			(13, 21, 22)

*Related substances, NO (Nitric Oxide), EDRF (endothelium-derived relaxing factor), VCAM (vascular cell adhesion molecule)-1, MIF (Macrophage migration inhibitory factor), IL (Interleukin)-1β, 6, 8, PGE (Prostaglandin)-2, TNF (Tumor Necrosis Factor)-α, COX (cyclooxygenase)-1,2, MCP (monocyte chemoattractant protein)-1, TGF (transforming growth factor)-β1, MIP-2 (macrophage inflammatory protein), LDL (Low Density Lipoprotein), CGRP (calcitonin gene-related peptide)*.

*Related pathways: TNF (Tumor Necrosis Factor), NF-kappa B (nuclear factor-kappa B), VEGF (vascular endothelial growth factor), PI3K/AKT/mTOR (phosphatidylinositol 3-kinase (PI3K)/AKT/ mammalian target of rapamycin (mTOR)*.

### The Vasodilatory Effect by the Increasing NO Production

Tomita, et al. visualized the immediate vasodilatory effect of KBG to investigate “peripheral circulatory insufficiency” -one pathological aspect of “blood stasis.” KBG induced significant vasodilation and improved blood velocity in arterioles of murine subcutaneous vessels detected by live imaging technics. This vasodilation peaked 60 min after administration and persisted for 90 min (2). The visualized image of rat mesenteric arterioles after KBG administration was evaluated using erythrocyte congestion, broadening the cell free layers as the pathology of the “blood stasis.” This study revealed an increase of nitric oxide, an endothelium-derived relaxing factor (EDRF), in the arterial endothelium of rat mesenteric arteries, especially at bifurcations, following KBG administration by live imaging (2). They concluded that the vasodilatory effect of KGB is due to the increasing NO production in the vascular endothelial cells.

### Protective Effect Against NO-Induced Neurotoxicity and Inflammation

Other than the vasodilatory effect of Nitric Oxide by eNOS, NO could cause cytotoxicity. Shimada, et al. reported that KBG was effective against NO-induced neurotoxicity caused by activation of neuronal NO synthase (nNOS). KBG had protective effect against NO-mediated neuronal death in cultured cerebellar granule cells and its effect is derived from Cinnamomi Cortex, Paeoniae Radix and Moutan Cortex (14). The association between the inhibitory effect of KBG on inflammatory cytokines and NO has been reported. Yoshihisa et al. evaluated the role of KBG in inhibiting the inflammatory cytokines using human dermal microvessel endothelial cells. KBG as well as paeoniflorin treatment significantly suppressed the mRNA levels of migration inhibitory factor (MIF), IL-6, 8 and tumor necrosing factor-α in LPS stimulated cultured human dermal microvessel endothelial cells. ELISA also showed KBG as well as paeoniflorin suppressed the production of these cytokines. In addition, KBG and paeoniflorin suppressed the expression of cyclooxygenase-2 and inducible nitric oxide synthase(iNOS) in these cells (15). This study suggested that KBG showed inhibitory effect on inflammatory cytokines by suppression against iNOS in skin endothelial cells and its mechanism of action.

### Protective Effect on the Endothelial Function Against Oxidative Stress

Diacron-reactive oxygen metabolites (d-ROMs) is a simple test for measuring oxidative stress in plasma. The antioxidant activity of Kampo prescription using d-ROMs had also been reported. Keishibukuryogan-ka-yokuinin, which is KBG plus one herbal medicine, yokuinin, which is coix seeds (*Coix lacryma-jobi var. ma-yuen*), decreased level of the d-ROMs in plasma. Reactive oxygen species (ROS)-scavenging and lipid hydroperoxide generation assays revealed that gallic acid, 3-O-methylgallic acid, (+)-catechin, and lariciresinol possess strong antioxidant activities (5). Nozaki, et al. reported the KBG's protective effect of vascular function by anti-oxidative effect. This study reported that KBG protected the endothelial function of Adjuvant-induced arthritis (AIA) rats mainly by its anti-oxidative effect. KBG improved endothelium-dependent relaxation by acetylcholine in the AIA and decreased the contractions by xanthine oxidase, plasma level of lipid oxidase and restricted increase of the expression of endothelial NO synthase, inducible NO synthase (iNOS) and VCAM-1 of thoracic aorta (3). Other than KBG, many herbal prescriptions are known to possess beneficial effects against oxidative stress (6).

### Protective Effect on RBC Against Oxidative Stress

Sekiya et al. reported that KBG provided a protective effect against the fragility of erythrocyte cell membranes caused by active oxygen (7). This study was an attempt to explain the concept of blood stasis by the relationship between active oxygen and red blood cells in the blood. Since blood stasis was also defined as “red blood's stasis” in medical classics in China, it is possible that the pathophysiology of red blood cells and blood stasis is related. Sekiya, et al. reported that KBG provided strong protection for RBC membranes against haemolysis induced by 2,2-azo-bis (2-amidinopropane) dihydrochloride, an azo free radical initiator. Inhibitory effect was dose dependent at concentration of 100–1,000 microg/ml. Furthermore, ingestion of 200 mg of KBG was associated with a significant decrease in susceptibility of RBC to haemolysis in rats (7). Another study of the pathophysiology of “blood stasis” about RBC, though in humans, reported that not only erythrocyte aggregability but also deformability was related to the “blood stasis” (23).

### Inhibitory Effect on Platelet Aggregation

The relationship between coagulopathy, hyperviscosity and “blood stasis” was studied by the effect of KBG on anti-platelet aggregation. The platelet aggregation was measured by pressure rate and the platelet aggregatory threshold index (PATI) values on collagen-induced platelet aggregation of guinea pig whole blood. Significant difference was observed in 1,000 μg/mL-KGB group (*P* < 0.0001) compared to control group. KBG inducement suppressed the collagen-induced whole blood pressure rate increase and increased the PATI value. Focusing on the herbal ingredients of KBG, paeonol, a representative component of Moutan cortex, and aspirin which is known to have platelet aggregation-inhibitory activity (COX-1 inhibitor) also showed similar effects. Based on these results, they suggested that the platelet aggregation-inhibiting activity of the constituent crude drug Moutan cortex and Cinnamomi cortex is involved in the improved effects of KBG on impaired microcirculation and that paeonol plays a role in these effects (4).

### Anti-inflammatory Effect by Decreasing COXs' Expression

Zhang et al. evaluated the anti-inflammatory functions of KBG, as well as its major ingredients, in human umbilical vein endothelial cells (HUVECs). The application of KBG significantly downregulated the mRNA expressions of cyclooxygenase-1 (COX-1) and cyclooxygenase-2 (COX-2) mRNAs in dose-dependent manner. Nine major components of KBG were tested in the inflammatory system, and three compounds-paeoniflorin, benzoylpaeoniflorin, and amygdalin-exhibited robust activation in HUVECs. The combination of paeoniflorin, benzoylpaeoniflorin, and amygdalin showed over 80% of the anti-inflammatory activation (8).

Another study reported that compared with LPS (Lipopolysaccharide) treated group, KBG and its active complex dose-dependently reduced the releasing of IL-1β, TNF-α and Prostaglandin E2 (PGE2) induced by LPS in RAW264. Seven cells. Moreover, the expression of IL-1β and microsomal prostaglandin E2 synthase-1 (mPGES-1) was decreased after KBG and its active complex treatment, which might contribute to the inhibitory effect of KBG in the releasing of IL-1β, TNF-α and PGE2 (9).

### Nephroprotective Effect

There are studies that report its efficacy as prevention of progression of chronic renal failure. Based on the effect of KBG against kidney of 5/6 nephrectomized rats, a well-characterized model of chronic renal failure (CRF), Nakagawa et al. reported that KBG exerts beneficial effects that results in slowing the progression of CRF (16). This study investigated the following effects of KBG against I) macrophage infiltration, II) accumulation of extracellular matrix (ECM), and III) oxidative stress, all of which are considered to be the pathogenesis of the development of CRF.

#### Inhibitory Effect on Macrophage Infiltration

Macrophage infiltration is evaluated by osteopontin, MCP-1 and VCAM-1 mRNA levels. The administration of KBG significantly suppressed osteopontin, while it showed a tendency to decrease MCP-1 and VCAM-1 mRNA levels without statistical significance. Osteopontin, potent chemotactic and adhesion molecule for monocyte/macrophage, has been shown to have strong association with focal macrophage infiltration in a number of experimental models of renal injury, suggesting a pathologic role in progressive renal injury. They concluded that these results suggest that KBG inhibits macrophage infiltration by suppression of mRNA levels related to macrophage infiltration (16).

#### Inhibitory Effect of Accumulation of Extracellular Matrix

Accumulation of ECM proteins, such as fibronectin, type IV collagen and laminin, conspicuous finding accompanying the progression of renal failure, is one of the pathogeneses of progressive renal failure. TGF-beta1 has been implicated as playing central role in the regulation of the over- deposition of ECM proteins. KBG treatment significantly suppressed mRNA levels of TGF-beta1 and fibronectin, suggesting that KBG exerts beneficial effects on the kidney by inhibiting ECM protein accumulation accompanying the progression of CRF induced by TGF-beta (16).

#### Inhibitory Effect Against Oxidative Stress

KBG administration showed significant reduction in serum urea nitrogen and urinary protein, not creatinine, compared with the control group. Oxidative stress is widely recognized to involve the pathogenesis of CRF. They speculated that KBG improved oxidative condition and microcirculation in the kidney of CRF, and these effects may contribute, at least in part, to the attenuation of serum urea nitrogen and urinary protein excretion, not serum creatinine. They reported in another article that KBG decreased lipid peroxidation and elevated superoxide dismutase activity in the kidney (18, 24). Their speculation about significant reduction in serum urea nitrogen and urinary protein by KBG was based on their previous research about KBG's oxidative effect. The effect of KBG on serum creatinine was unchanged (16) and reduced (24). Nakagawa et al. reported that oral administration of KBG in spontaneous diabetic WBN/Kob rats significantly attenuated urinary protein excretion and serum creatinine level. KBG also reduced fibronectin and TGFβ1 protein expression of the renal cortex. Furthermore, lipid peroxidation levels in both kidney and liver were significantly lower than those of untreated control WBN/Kob rats. Urinary expression of 8-hydro-deoxyguanosine, an oxidative stress marker, was suppressed by KBG treatment. These results suggest that KBG reduces oxidative stress by hyperglycemia, and it protects renal function and suppresses fibronectin deposition induced by TGFβ1 production in WBN/Kob rats (24).”

### The Effect of KBG on Renal Transporter as Nephroprotective Agents

Lee S.H. et al. hypothesized that KBG may modulate the renal transporter function, URAT1, OAT1 and OAT3, which are responsible for the renal reabsorption of uric acid and mediate the renal uptake of organic anions, drugs, and metabolic toxins, as the primary contributors to the drug-induced nephrotoxicity (19). They reported that KGB inhibited the substrate uptake activities of renal transporter, the urate transporter 1 (URAT 1), the organic anion transporters (OAT1 and OAT 3) in Xenopus oocyte and HEK 293 human kidney embryonic cells, suggesting their mechanism of action as nephroprotective agents. OAT-organic anion transporter-membrane proteins that mediate the translocation of diverse compounds across biological membranes, occupy the largest portions in the regulation of kidney physiological processes. About the relationship between KBG with renal transporters, further research is expected on the specific renal protective effect of KBG.

### Regulation of Systemic Adipocytokines Level and/or Lipid Metabolism

The following reports suggested that KBG could improve obese status through a regulation of systemic leptin level and/or lipid metabolism. Adipocyte hypertrophy and adipocytokines, such as leptin and TNF-α, are considered to be key pathological contributors to insulin resistance.

Gao et al. reported that KBG treatment significantly decreased the serum level of leptin and liver triglyceride (TG) level in the diet-induced obesity mouse (10). In addition, a lower fat deposition in liver and a smaller size of adipocytes in white adipose tissue were observed in the diet-induced obesity mouse treated with KBG. They found downregulation of genes involved in lipid metabolism in the KBG-treated liver, along with decreased liver TG and cholesterol level (10).

Nakagawa et al. reported that KBG significantly lowered serum total cholesterol and triglyceride levels, and the hepatic total cholesterol, and reduced serum leptin level, not the serum adiponectin level in Otsuka Long-Evans Tokushima Fatty (OLETF) rats, an animal model of type 2 diabetes. They also showed significant effects of KBG on epididymal adipose tissue by decreasing the size of fat cells and on skeletal muscle by reducing TNF-α protein content, a crucial factor responsible for insulin resistance in obese and diabetic subjects (11). They suggested that KBG exerted a hypolipidemic effect in the diabetic body and this effect contributed to amelioration of the insensitivity of peripheral tissues to insulin and glucose disposal.

### Inhibiting the Progression of Atherosclerosis in Hypercholesterolemia by Antioxidant Effect

Sekiya et al. reported in their study of cholesterol-fed rabbits, that of the control, the KBG and vitamin E groups, the platelet counts at the end of the study were significantly lower than those determined prior to study and the progression of visible plaque was inhibited in KBG groups compared to the control (*P* < 0.01). The serum lipid peroxidase was significantly lower in KBG and vitamin E groups, compared with control group (*P* < 0.05) and urinary 8OHdG-an oxidative marker-was significantly lower in KBG group compared with vitamin E group (*P* < 0.05). These results in cholesterol-fed rabbits suggested that KBG prevented the progression of atheromatous plaque by creating a sounder antioxidant defense system than vitamin E as lipid soluble antioxidant (12). They previously reported that aortic surface involvement of control rabbits were greater than that of KGB treated rabbits statistically (*P* < 0.01), in both LDL and beta-VLDL, and lipid peroxide formation in KBG treated rabbits were less than that in control rabbits statistically (P <0.01). KBG suppressed the *in vitro* lipid peroxide formation dose-dependently compared with control. KBG administered rabbits showed the suppression of serum lipid peroxidase formation compared with rabbits before its administration (*P* < 0.05). Based on this result, they concluded that KBG prevents the progression of atherosclerosis in cholesterol-fed rabbits *in vivo* by limiting oxidative LDL modification (25).

### Inhibitory Effect on Fibrosis Against Oxidative Stress

Fujimoto et al. investigated that KBG could be a candidate to prevent the progression of non-alcoholic fatty liver disease (NAFLD) to steatohepatitis (NASH) in standard rabbits (26). The result suggested that KBG was superior to vitamin E and pioglitazone in the reduction of the liver total cholesterol (*P* < 0.01) and lipid peroxidase (*P* < 0.05), urinary 8-hydroxy-2' deoxyguanosine (*P* < 0.05), hepatic α-smooth muscle actin (α-SMA) positive areas (*P* < 0.05) and activated stella cells (*P* < 0.05). A-SMA is a specific marker for smooth muscle cell differentiation, which is related to the process of hepatic fibrosis, and is a reliable marker of hepatic stella cell activation which precedes fibrosis tissue deposition (17, 27, 28). They concluded that there was a statistically significant benefit of KBG, in particular, on a dietary model of NAFLD/NASH. Two-hit hypothesis is prevailing theory for the development of NAFLD. Oxidative stress is considered one of factors to cause “second hit.” In this study, the process of organic disease, a chronic qualitative change caused by oxidative stress could be considered as one of the pathological conditions of “blood stasis.”

### Anti-fibrotic Effect on Systemic Sclerosis

There were studies related to “sclerosis” as a chronic qualitative change of “blood stasis.” KBG could be regarded as a synergistic therapeutic option which takes pharmacological actions by affecting multiple signaling pathways and different molecules rather than a single pathway. System biologic approaches suggested that KBS could suppress the proliferation of fibroblasts and decrease the Th1 cytokines; TNF-α, MIP-2 and IL-6 (29). Another study investigated the effect of KBG on collagen production in scleroderma fibroblast culture. KBG significantly (*P* < 0.05) inhibited collagen product at each incubation time at 0, 6, 12 and 24 h, in a dose-dependent manner. A significant difference was demonstrated between the untreated and KBG-treated condition in each fibroblast line from three scleroderma patients. Sheng, et al. concluded that KBG significantly and selectively inhibited collagen synthesis in a dose-dependent manner, with a tendency of a stronger effect on scleroderma fibroblasts than control cells (30).

### The Effect on Endometrial Hyperplasia by Triggering Ferroptosis

The term ferroptosis was coined in 2012 to describe an iron-dependent regulated form of cell death caused by the accumulation of lipid-based reactive oxygen species (31). It is morphologically, biochemically, and genetically distinct from apoptosis, necrosis, and autophagy (32). Recent studies have shown that ferroptosis is closely related to the pathophysiological processes of many diseases, such as tumors, nervous system diseases, ischemia-reperfusion injury, kidney injury, and blood diseases (33).

Zhang et al. investigated the effect of KBG on endometrial hyperplasia from the viewpoint of ferroptosis. According to their research, the degree of ferroptosis in endometrial tissue of patients was lower than in normal endometrial tissue. In addition, ferroptosis inducer imidazole ketone erastin (IKE) could improve endometrial hyperplasia in mice. Interestingly, KBG significantly alleviated endometrial hyperplasia through triggering ferroptosis. Furthermore, in estradiol-induced endometrial hyperplasia model, KBG inhibited p62-Keap1-NRF2 pathway which is the major regulator of cytoprotective responses to oxidative and electrophilic stress and is related to increasing cancer chemoresistance and enhancing tumor cell growth (13, 21). They concluded that KBG may attenuate estrogen-induced endometrial hyperplasia in mice through triggering ferroptosis *via* inhibiting p62-Keap1-NRF2 pathway (13).

### Inhibitory Effects of Autophagy on Polycystic Ovary Syndrome

Polycystic ovary syndrome (PCOS) is a common reproductive and endocrinologic disorder and three main phenotype characteristics of this condition are hyperandrogenism, polycystic ovaries, and ovulatory dysfunction (22). This syndrome can also be associated with metabolic issues including obesity, insulin resistance (found in 60–80% of women with PCOS) (34). hyperinsulinemia, and type 2 diabetes mellitus (T2DM). The cause of PCOS remains largely unknown, but studies suggest an intrinsic ovarian abnormality such as granulosa cell survival and proliferation (20).

Liu et al. reported that KBG inhibited granulosa cell autophagy and promoted follicular development to attenuate ovulation disorder in PCOS-insulin resistance rats. They concluded that this result was associated with activation of the phosphatidylinositol 3-kinase (PI3K)/AKT/ mammalian target of rapamycin (mTOR) signaling pathway, which plays an important role in the regulation of cell survival, growth, and proliferation (20).

Das et al. reported that apoptosis is associated with the pathophysiology of PCOS (35). It is necessary to verify the effect of KBG on regulated cell deaths, such as apoptosis, necroptosis, and autophagy.

### The Possible Effect on Initiation and Growth, Related to Inflammation, Fibrosis, Proliferation, and Angiogenesis of Uterine Fibroids

Uterine leiomyomas (ULs), also called uterine fibroid, arise due to transformation of the layer of smooth muscle cells of corpus uteri. Despite frequent occurrence of this disease, the molecular mechanisms behind the origin and development of leiomyomas are still relatively unknown (36). However, dysregulation of inflammatory processes is thought to be involved in the initiation of leiomyoma, and the following extracellular matrix deposition-cell proliferation, and angiogenesis-are the key cellular events implicated in leiomyoma growth (37).

Based on TCM, ULs belong to the concept of “Zhengjia” and “Jiju”, which mean that they are caused by “blood stasis” and “Qi stagnation” (38).

Li et al. reviewed the effect of KBG on ULs. Among a total of 21 studies (22 experiments) involving 461 female animals, including guinea pig (*n* = 20), rats [*n* = 385, Sprague-Dawley (SD) and Wister] and mice (*n* = 56), the available evidence suggests that KBG has potentially beneficial effects over placebo on both fibroid characteristics and sex hormones in SD rats (except progesterone), Wister rats (except progesterone receptor gene expression) and Institute of Cancer Research (ICR) mice (except PR gene expression). KBG appears to reduce uterine weight and smooth muscle thickness in the guinea pig, but no data on sex hormone index was available. They concluded that that KBG may be a promising intervention for the management of uterine fibroids in animal models (39).

The effect of KBG may be related to the processes of initiation and growth, related to inflammation, fibrosis, proliferation, and angiogenesis, of ULs. Further research is expected in the future.

### The Effect on Female Hormonal Dynamics

There are various results about the effect of KBG on female hormone dynamics, however, these results were very controversial.

Usuki reported several articles about the effect of KBG on hormone dynamics. One of them showed that KBG decreased estradiol-17 beta (E2) levels in media and luteinizing hormone (LH) effects on progesterone secretions, while they increased progesterone in media and LH effects on E2 secretions in rats preovulatory follicles. He concluded that KBG stimulated preovulatory follicles to secrete progesterone but to suppress E2 secretions, and it was indicated that their combination treatment with LH multiplies the sole effect of LH on E2 secretions but suppresses LH effects on progesterone (40).

Another article reported that the concentrations of E2 were significantly decreased with KBG by rat growing follicles and preovulatory follicles before a LH surge. In contrast, the levels of progesterone significantly increased with KBG by preovulatory follicles before a LH surge. These results suggested that KBG stimulated preovulatory follicles before a LH surge to secrete progesterone, but that KBG suppresses E2 secretion by growing preovulatory follicles before a LH surge (41). However, he showed different results in ovarian tissue from pregnant mare serum gonadotropin (PMS)-treated immature rats as mentioned before. He reported that administration of KBG increased the concentrations of E2, progesterone and testosterone. He concluded that KBG stimulated *in vivo* the production of E2, progesterone and testosterone by preovulatory follicles (42).

Sakamoto, et al. reported that long-term daily oral administration of KBG (300 mg/kg) in rats for 14 days decreased plasma levels of LH, follicle stimulating hormone (FSH) and E2 by 94%, 67% and 64% that of controls, respectively and KBG enhanced luteinizing hormone-releasing hormone (LH-RH)-induced increase in plasma LH and FSH levels 1.2- and 2.5-fold respectively, as compared with controls. He concluded that the results indicate that KBG may act as a LH-RH antagonist and /or a weak anti-estrogen (43). Wang et al. evaluated the estrogenic activity of these five herbal medicines and their metabolites using an estrogen receptor-dependent bioassay and an estrogen receptor-dependent reporter assay, and suggested that KBG did not exert estrogenic activity (44).

### The Effect on Hot Flash by Normalizing the Attenuated CGRP Release Process

Hot flash, which is one of the symptoms of menopausal syndrome, might be described as a “the blood stasis of head and upper body.” The physiological changes associated with the hot flash are different from any other flushing condition, i.e., an increased peripheral blood flow, increased heart rate, and in particular a decrease in galvanic skin resistance, which is unique to the flash (45). The evidence of various recent studies supports the role of calcitonin gene-related peptide (CGRP) as a predominant neurohormone involved in vasomotor symptoms and possibly are due to a release of this vasodilatory peptide, CGRP, from perivascular nerves (46). CGRP could act centrally on the thermoregulatory center of the hypothalamus as well as peripherally to cause vasodilation and sweating (47).

Chen et al. evaluated the relationship between CGRP and the effect of KBG on menopausal hot flash. The result was that not vasoactive intestinal peptide (VIP) but plasma CGRP, significantly elevated at the occurrence of hot flash (*P* = 0.002). Stress by cold load also significantly enhanced the over-secretion of CGRP in subjects with hot flash compared with those without hot flash (*P* = 0.003) 3 min after the load. KBG decreased plasma CGRP level in subjects with hot flash. They concluded that not vasoactive intestinal peptide (VIP) but CGRP was mainly related to the occurrence of hot flash and that KBG improves hot flash possibly by affecting plasma CGRP level (48).

Ovariectomy also triggers menopausal symptom. Ovariectomy not only potentiated CGRP-induced elevation of skin temperature and arterial vasorelaxation but also induced a lower concentration of endogenous CGRP in plasma and up-regulation of arterial CGRP receptors. It suggests that lowered CGRP in plasma due to ovarian hormone deficiency increases the number of CGRP receptors and consequently amplifies the stimulatory effects of CGRP to elevate skin temperature.

Noguchi et al. investigated the effects of 17 beta-estradiol (E2) and KBG on the release and synthesis of CGRP in ovariectomized (OVX) rats (49). Oral KBG (100–1,000 mg/kg, once a day for 7 days) restored a series of CGRP-related responses observed in OVX rats by normalizing plasma CGRP levels in a dose-dependent manner as effectively as subcutaneous injection of E2 (0.010 mg/kg, once a day for 7 days). However, KBG did not affect the lower concentration of plasma estradiol and the decreased uterine weight due to ovariectomy, although the hormone replacement of 17 beta-estradiol restored them. They concluded that these results suggested that KBG, which does not confer estrogen activity on plasma, may be useful for the treatment of hot flashes in patients for whom estrogen replacement therapy in contraindicated, as well as menopausal women (49).

Noguchi et al. also investigated the effects of E2 and KBG on the release and synthesis of CGRP in OVX rats. Ovariectomy attenuated the capsaicin-evoked increase in plasma concentration of CGRP, which was restored by treatment with subcutaneous E2 injection or KBG for 7 days after ovariectomy. However, no significant differences were observed in the CGRP concentration and the mRNA expression of dorsal root ganglia-which synthesized endogenous CGRP-in OVX rats by treating with E2 and KBG. These results suggest not only that estrogen deficiency attenuates CGRP release, but also that E2 or KBG normalizes the attenuated CGRP release process (50).

### Network Pharmacology Approaches

In 2007, Hopkins created a novel concept of network pharmacology, which is built on the fundamental concept that many effective drugs in therapeutic areas act on multiple rather than single targets (51, 52). With this concept, network pharmacology can be reconstructed with molecular networks that integrate multidisciplinary concepts including biochemical, bioinformatics, and systems biology (53). In China especially, network pharmacology is increasingly applied in TCM formula research in recent years, which is identified as suitable for the study of TCM formula. Network pharmacology has become a helpful tool to achieve the interaction between the bioactive compounds and targets and the interaction between various targets, and then find out and validate the key nodes *via* network analysis and network verification. This is especially useful for multiple drug components of Chinese Herbal Medicine (54, 55).

Wang et al. reported that the results of network pharmacology research supported the premise that the potential mechanism of KBG in the treatment endometriosis might be inflammatory pathways, such as, TNF signaling pathway (*P* < 0.01) and NF-kappa B signaling pathway (*P* < 0.01) (56).

Network pharmacologic approach also indicated that the “anti-sclerotic” effect of KBG involved vascular endothelial growth factor (VEGF) signaling pathway, which relates to the process of scleroderma microvasculature, and the Toll-like receptor signaling pathway, which is a pro-fibrotic process of scleroderma (29). Since herbal medicines are composed of multiple crude drugs and have various mechanisms of action, a research method for grasping the whole picture by network pharmacology may be useful, but it is still under research.

## Clinical Research

The following clinical research would be useful to understand the efficacy of KBG and “blood stasis.” Although KBG is frequently used in cardiovascular surgery and gynecology (57), it is also used in the other departments to treat diseases related to “blood stasis.” Clinical research of KBG would show that “blood stasis” might be related to the function of vascular endothelium and red blood cell deformability, and the drug could improve varicose vein, hematoma, deep vein thrombosis, rheumatoid arthritis, atopic dermatitis with lichenification, sensory symptom, hot flash, and other symptoms in pre- and postmenopausal women.

### Function of Vascular Endothelium and Red Blood Cell Deformability

Nagata et al. reported that KBG improved vascular endothelial function assessed by reactive hyperemia peripheral arterial tonometry, reduced malondialdehyde which is a marker for oxidative stress and decreased serum non-esterified fatty acid in patient with metabolic syndrome related factors by controlled clinical trial (58). “Blood stasis” would be related to endotherial function, oxidative stress and arteriosclerosis induced by non-esterified fatty acid. On the other hand, Hikiami et al. reported that in patients with multiple lacunar infarctions, KBG had significant effects on red blood cell deformability as evaluated by filtration method (23), which might suggest that “blood stasis” would be related to deformation of blood cell.

### Varicose Vein, Hematoma and Deep Vein Thrombosis

There are also the following reports related to the imaging of improved blood flow. Hayashi et al. reported that in patient of varicose veins of the lower extremity, KBG could improve subjective symptoms and severity of varicose veins, decrease score of “blood stasis,” and increase skin perfusion pressure. The effect especially was remarkable in female (59). The concept of blood stasis also involved hematoma and deep vein thrombosis. In university hospital, KBG was used for treating hematoma in emergency department (60). Kumanomido et al. reported that KBG diminished subcutaneous hematoma after surgery in a case report (61). RCT also suggests that in elderly subjects, KBG improved deep vein thrombosis of lower limb (62).

### Rheumatoid Arthritis

Nozaki et al. reported that in patients with rheumatoid arthritis, KBG showed decreased disease activity against modified disease activity score, reduced soluble vascular adhesion molecule-1 which has been postulated to be useful risk predictors of the progression of atherosclerosis and cardiovascular events, and decreased lipid peroxide. However, it did not alter CRP, IL-1β, IL-6 and TNF-α (63). This report showed that KBG might decline disease activity of rheumatoid arthritis through an antioxidative action and the prevention of atherosclerosis apart from anti-inflammatory function.

### Atopic Dermatitis

The findings of fixed hard skin such as skin lesion of lichenification are also one of the criteria for blood stasis in traditional medicine. Mizawa et al. reported that KBG improved lichenification in patients of atopic dermatitis and was more effective in those having high lichenification score. On the other hand, KBG decreased LDH but did not change serum IgE and blood eosinophils, which would suggest that this drug may improve lichenification through preventing tissue injury without altering factor related to allergies (64).

### Sensory Symptom

Fujita et al., reported that in patient who complained of cold sensation and numbness after cerebral stroke, KBG improved both cold sensation and numbness with visual analog scale, and increased skin temperature of diseased limb whereas did not change that of healthy limb (65). KBG might normalize sensory system or blood flow.

### Hot Flash

KBG is one of the most used treatment in department of obstetrics and gynecology division. In particular, a number of reports showed KGB would be effective for hot flash. It is reported that KBG improved symptom of hot flash in young female assessed by visual analog scale (66). Moreover, Ushiroyama et al. reported that the administration of KGB decreased the blood flow under the jaw which would show signs of flash in the upper body and increased the blood flow in the lower extremities in postmenopausal women with hot flash (67). KBG was reported to be effective for hot flashes not only in female but also in male. Shigehara et al. reported that in prostate cancer patients receiving androgen deprivation therapy, KBG significantly reduced hot flash intensity, frequency and duration without the changes of prostate specific antigen and total testosterone level (68). It could be said that KBG would be effective for hot flashes related to sex hormone changes regardless of gender. On the other hand, another study reported that KBG could not change the degree of hot flash in postmenopausal women in randomized double-blind, placebo-controlled trial (69). In connection with hormonal metabolism to hot flash, Saruwatari et al. showed that KBG reduced CYP1A2, which is predominantly involved in estrogen metabolism (70). Yasui et al. reported that KBG reduced the circulating IL-8 and monocyte chemotactic protein-1 level in women with hot flashes (71). These changes of circulating hormone and chemokine might be related to improvement of the hot flash.

### Endometriosis

In department of gynecology, KGB was reported to be effective to treat endometriosis (EMs). There were some reports in which KGB in combination with western medicine had achieved satisfactory curative effects. Sun XL et al. reported that KGB combined with danazol and gestrinone capsules for treating patient of EMs could reduce the immune inflammatory response in ectopic lesions and inhibit angiogenesis, and the mechanism is related to the down-regulation of VEGF and Hypoxia Inducible Factor (HIF)-1α expression (72). KBG could inhibit cell proliferation and differentiation of endometriosis, by inhibiting the MAPK/ERK kinase (MEK) and extracellular signal-regulated kinase (ERK) protein activity, blocking the cell signaling pathways, which suggesting that KBG prevented tumor growth and differentiation (73). In randomized controlled trial, KGB assisted western medicine treatment had improved the clinical symptoms and signs, and quality of life of patients of EMs, of which mechanism can be related to its inhibition of serum Leptin, VEGF, IL-8 levels and improvement of ovarian function (74). By then, KBG and mifepristone had better clinical effects on the EMs, which could reduce the serum levels of cancer antigen (CA)125 and CA199 (75). Moreover, Qian J et al. reported that the treatment using danazol with KGB had more long-lasting effect and lower recurrence rate in patients of EMs than that of danazol alone (76). KGB in combination use with western medicine was considered to be useful treatment candidate of EMs.

### Others

KBG could change some symptoms of postmenopausal syndrome such as improved subjective sleep disturbances, alleviated perspiration, and reduced systolic/diastolic pressure, in pre- and postmenopausal women (77). In the premenopausal patients with uterine myomas, KBG improved clinical symptoms of hypermenorrhea and dysmenorrhea with shrinking of uterine myomas (78). All these symptoms may be related to the traditional concept of “blood stasis.” Especially in Kampo-related diagnosis of “blood stasis” in clinical situation, an abdominal examination called Fukushin is fundamental skill. Yakubo et al. recently developed a clinical simulator of the examination and Arita et al. put it into practice in medical education (79, 80). Anatomical analysis of the findings of “blood stasis,” such as lower abdominal resistance and fullness on abdominal examination, and its relationship with KBG could be an issue for the future.

These clinical research would suggest that KBG was applicable to various pathological conditions and changed many kinds of circulating factors seemed to be related to the mechanism, which could bridge the traditional concept of “blood stasis” and modern science.

## Limitation

In this review, we focused on summarizing the results of basic and clinical studies so far and gaining an overview of the action of KBG. One of the limitations is that the concrete presentation of future research could not be fully shown in figures and roadmaps.

## Conclusion

In basic research, the effects of Keishibukuryogan (KBG)/ gui-zhi-fu-ling-wan (GFW), one of the most common anti- “blood stasis” prescriptions, indicated that the initiation of quantitative changes closely were related to loss of redox balances on endothelial function induced by oxidative stress, and that the following qualitative changes were related to coagulopathy, hyper viscosity; anti-platelet aggregation, lipid metabolism; regulation of systemic leptin level and/or lipid metabolism, inflammatory factor; COX-1, 2, IL-6, 8 TNF-α, macrophage infiltration, hyperplasia, tissue fibrosis and sclerosis caused by TGF-β1 and fibronectin, dysfunction of regulated cell deaths such as, apoptosis, autophagy, ferroptosis and ovarian hormone imbalance.

Clinically, KBG, was often used for diseases related to Obstetrics and Gynecology, Endocrine Metabolism, Rheumatology and Dermatology.

In basic research, a re-verification through additional examinations of existing research and other research designs will be necessary. Since herbal medicines are composed of multiple crude drugs and have various mechanisms of action, a research method for grasping the whole picture by network pharmacology is expected to give us a bird's-eye view to provide a broader spectrum image and clarify the pathophysiology of KBG and “blood stasis.” KBG is frequently used in clinical practice, and some have clear indications. Further clinical research on the prescription for “blood stasis” containing KBG is necessary based on the results of basic research.

## Author Contributions

KT and KC contributed to conception and writing of the manuscript. KN critically revised the manuscript. All authors contributed to this manuscript and approved the final manuscript.

## Conflict of Interest

The authors declare that the research was conducted in the absence of any commercial or financial relationships that could be construed as a potential conflict of interest.

## Publisher's Note

All claims expressed in this article are solely those of the authors and do not necessarily represent those of their affiliated organizations, or those of the publisher, the editors and the reviewers. Any product that may be evaluated in this article, or claim that may be made by its manufacturer, is not guaranteed or endorsed by the publisher.

## References

[1] Perk B You S Jung J Lee KA Yun JK Lee MS. Korean studies on blood stasis: an overview. Evid Based Complement Alternat Med. (2015) 2015:316872. 10.1155/2015/316872 25821483 PMC4363678

[2] Tomita T Matsui H Aoyagi K. Effect of Keishibukuryogan, a Japanese traditional Kampo prescription, on improvement of microcirculation and Oketsu and induction nitric oxide; a live imaging study. Evid Based Complement Alternat Med. (2017) 2017:3620130. 10.1155/2017/3620130 28785289 PMC5530408

[3] Nozaki K Goto H Nakagawa K Hikiami H Koizumi K Shibahara N. Effects of Keishibukuryogan on vascular function in adjuvant-induces arthritis rats. Biol Pharm Bull. (2007) 30:1042–7. 10.1248/bpb.30.1042 17541151

[4] Terawaki K Noguchi M Yuzurihara M Omiya Y Ikarashi Y Kase Y. Keishibukuryogan, a traditional Japanese medicine, inhibits platelet aggregation in Gunia pig whole blood. Evid Based Complement Alternat Med. (2015) 1–9. 10.1155/2015/295706 26379740 PMC4561328

[5] Matsubara Y Matsumoto T Sekiguchi K Koseki J Kaneko A Yamaguchi T. Oral administration of Japanese traditional medicine Keishibukuryogan-ka-yokuinin decreases reactive oxygen metabolites in rat plasma: identification of chemical constituents contributing to antioxidant activity. Molecules. (2017) 22:256. 10.3390/molecules22020256 28208738 PMC6155852

[6] Nishimura K Osawa T Watanabe K. Evaluation of oxygen radical absorbance capacity in Kampo medicine. Evid Based Complement Alternat Med. (2011) 1–7. 10.1093/ecam/nen082 19126557 PMC3137646

[7] Sekiya N Goto H Shimada Y Terasawa K. Inhibitory effects of Keishi-bukuryo-gan on free radical induced lysis of rat red blood cells. Phytother Res. (2002) 16:373–6. 10.1002/ptr.757 12112296

[8] Zheng Y Xin G Gong G Dong T Li P Tsim WK. Evaluation of anti-inflammatory components of Guizhi Fuling capsule, an ancient Chinese herbal formula, in human umbilical vein endothelial cells. Evid Based Complement Alternat Med. (2020) 2020:2029134. 10.1155/2020/2029134 33149750 PMC7603573

[9] Zhang Z Zhang X Li N Cao L Ding G Wang Z. Study on anti-inflammation effect and involved mechanism of Guizhi Fuling capsule and its active complex. Zhongguo Zhong Yao Za Zhi. (2015) 40:993–8. 26226733

[10] Gao F Yokoyama S Fujimoto M Tsuneyama K Saiki I Shimada Y Hayakawa Y. Effect of Keishibukuryogan on genetic and dietary obesity mouse. Evid Based Complement Alternat Med. (2015) 1–8. 10.1155/2015/801291 25793003 PMC4352422

[11] Nakagawa T Goto H Hussein G Hikiami H Shibahara N Shimada Y. Keishibukuryogan ameliorate glucose intolerance and hyperlipidemia in Otsuka long-evans tokushima fatty (OLETF) rats. Diabetes Res Clin Pract. (2008) 80:40–7. 10.1016/j.diabres.2007.11.019 18242756

[12] Sekiya N Kainuma M Hikiami H Nakagawa T Kouta K Shibahara N. Oren-gedoku-to and Keishi-bukuryo-gan ryo inhibit the progression of atherosclerosis in diet induced hypercholesterolemic rabbits. Biol Pharm Bull. (2005) 28:294–8. 10.1248/bpb.28.294 15684487

[13] Zhang M Zhang T Song C Qu J Gu Y Liu S. Guizhi Fuling Capsule ameliorates endometrial hyperplasia through promoting p62-Keap1-NRF2-mediated ferroptosis. J Ethnopharmacol. (2021)274:114064. 10.1016/j.jep.2021.114064 33771639

[14] Shimada Y Yokoyama K Goto H Sekiya N Mantani N Tahara E. Protective effect of Keishi-bukuryo-gan, and its constituent medicinal plants against nitric oxide donor-induced neuronal death in cultured cerebellar granule cells. Phytomedicine. (2004) 5:404–10. 10.1016/j.phymed.2003.04.002 15330495

[15] Yoshihisa Y Furuichi M Rehman MU Ueda C Makino T Shimizu T. The traditional Japanese formula Keishibukuryogan inhibits the inflammatory by dermal endothelial cells. Mediators Inflamm. (2010) 2010:804298. 10.1155/2010/804298 21253500 PMC3021874

[16] Nakagawa T Tashiro I Fujimoto M Jo M Sakai S Oka H. Keishibukuryogan reduces renal injury in the early stage of renal failure in the remnant kidney model. Evid Based Complement Alternat Med. (2011) 2011:914249. 10.1093/ecam/nep089 19633031 PMC3137790

[17] Nouchi T Tanaka Y Tsukada T Sato C Marumo F. Appearance of alpha-smooth-muscle-actin-positive cells in hepatic fibrosis. Liver. (1991) 11:100–5. 10.1111/j.1600-0676.1991.tb00499.x 2051901

[18] Nakagawa T Goto H Hikiami H Yokozawa T Shibahara N Shimada Y. Protective effects of Keishibukuryogan on the kidney of spontaneously diabetic WBN/Kob rats. J Ethnopharmacol. (2011) 110:311–7. 10.1016/j.jep.2006.09.043 17123761

[19] Lee HS Shin HJ Cho MSH Lee SH Oh D-S. Inhibitory effects of Kampo medicines, Keishibukuryogan and Syakuyakukanzoto, on the substrate uptake activities of solute carrier organic anion transporter. J Pharmacol Sci. (2018) 138:279–83. 10.1016/j.jphs.2018.10.008 30424926

[20] Liu M Zhu H Zhu Y Hu X. Guizhi Fuling Wan reduces autophagy of granulosa cell in rats with polystic ovary syndrome via restoring the PI3K/AKT/mTOR signaling pathway. J Ethnopharmacol. (2021) 270:113821. 10.1016/j.jep.2021.113821 33460753

[21] Kansanen E Kuosmanen SM Leinonen H Levonen AL. The Keap1-Nrf2 pathway: Mechanisms of activation and dysregulation in cancer. Redox Biol. (2013) 1:45–9. 10.1016/j.redox.2012.10.001 24024136 PMC3757665

[22] Rotterdam ESHRE/ASRM-Sponsored PCOS Consensus Workshop Revised 2003 consensus on diagnostic criteria and long-term health risks related to polycystic ovary syndrome (PCOS). Hum Reprod. (2004) 19:41–7. 10.1093/humrep/deh098 14688154

[23] Hikiami H Goto H Sekiya N Hattori N Sakakibara I Shimada Y. Comparative efficacy of Keishi-bukuryo-gan and pentoxifylline on RBC deformability in patients with “Oketsu” syndrome. Phytomedicine. (2003) 10:459–66. 10.1078/094471103322331395 13678228

[24] Nakagawa T Yokozawa T Oowada S Goto H Shibahara N Shimada Y. Amelioration of kidney damage in spontaneously diabetic WBN/Kob rats after treatment with Keishibukuryogan. J Trad Med. (2003) 20:156–64.

[25] Sekiya N Tanaka N Itoh T Goto H Terasawa K. Keishi-bukuryo-gan prevents the progression of atherosclerosis in cholesterol-fed rabbit. Phytother Res. (1999) 13:192–6. 10353155 10.1002/(SICI)1099-1573(199905)13:3<192::AID-PTR412>3.0.CO;2-2

[26] Fujimoto M Tsuneyama K Kainuma M Sekiya N Goto H Takano Y. Evidence-based efficacy of Kampo formulas in a model of non-alcoholic fatty liver. Exp Biol Med. (2008) 233:328–37. 10.3181/0707-RM-207 18296738

[27] Skalli O Ropraz P Trzeciak A Benzonana G Gillessen D Gabbiani G. A monoclonal antibody against alpha-smooth muscle actin: a new probe for smooth muscle differentiation. Cell Biol. (1986) 103:2787–96. 10.1083/jcb.103.6.2787 3539945 PMC2114627

[28] Carpino G Morini SSG Corradini A Franchitto M Merli M Siciliano. (2005). Alpha-SMA expression in hepatic stellate cells and quantitative analysis of hepatic fibrosis in cirrhosis and in recurrent chronic hepatitis after liver transplantation. Dig Liver Dis. 37:349–56. 10.1016/j.dld.2004.11.009 15843085

[29] Wang Q Shi G Zhang Y Lu F Xie D Wen C. Deciphering the Potential Pharmaceutical Mechanism of Gui-Zhi-Fu-Ling-Wan on Systemic Sclerosis based on Systems Biology Approaches. Sci Rep. (2019) 9:355. 10.1038/s41598-018-36314-2 30674993 PMC6344516

[30] Sheng FY Ohta A Yamaguchi M. Inhibition of collagen production by traditional Chinese herbal medicine in scleroderma fibroblast cultures, Internal Med. (1994) 33:466–71. 10.2169/internalmedicine.33.466 7803912

[31] Hirschhorn T Stockwell BR. The development of the concept of ferroptosis. Free Radic Biol Med. (2019) 133:130–43. 10.1016/j.freeradbiomed.2018.09.043 30268886 PMC6368883

[32] Dixon SJ Lemberg K Lamprecht M Skouta MR Zaitsev R Gleason EMCE. Ferroptosis: an iron-dependent form of nonapoptotic cell death. Cell. (2012) 1495:1060–72. 10.1016/j.cell.2012.03.042 22632970 PMC3367386

[33] Li J Cao F Yin H Huang ZJ Lin ZT Mao N. Ferroptosis: past, present and future. Cell Death Dis. (2020) 112:88. 10.1038/s41419-020-2298-2 32015325 PMC6997353

[34] Carmina E Oberfield SE Lobo RA. The diagnosis of polycystic ovary syndrome in adolescents. Am J Obstet Gynecol. (2013) 203:201.e1–201.e5. 10.1016/j.ajog.2010.03.008 20435290

[35] Das M Djahanbakhch O Hacihanefioglu B Saridogan E Ikram M Ghali L. Granulosa cell survival and proliferation are altered in Polycystic Ovary Syndrome. J Clin Endocrinol Metab. (2008) 933:881–7. 10.1210/jc.2007-1650 18073308 PMC2679149

[36] Dvorská D Braný D Danková Z Halašová E Višnovský J. Molecular and clinical attributes of uterine leiomyomas. Tumour Biol. (2017) 396:1010428317710226. 10.1177/1010428317710226 28653894

[37] Islam MS Akhtar MM Ciavattini A Giannubilo SR Protic O. Use of dietary phytochemicals to target inflammation. fibrosis, proliferation, and angiogenesis in uterine tissues: promising options for prevention and treatment of uterine fibroids? Mol Nutr Food Res. (2014) 58:1667–84. 10.1002/mnfr.201400134 24976593 PMC4152895

[38] Zhao H Li Y Xu Q Peng F Zhao JS Webb RC. Establishment of a rat model for uterine leiomyomas based on Western and traditional Chinese medicine theories. Braz J Med Biol Res. (2018) 519:e7627. 10.1590/1414-431x20187627 29972430 PMC6040866

[39] Li M Hung A Yang WAH. Guizhi Fuling Wan for uterine fibroids: A systematic review of in vivo studies. J Ethnopharmacol. (2019) 245:112177. 10.1016/j.jep.2019.112177 31445128

[40] Usuki S. Effects of hachimijiogan, tokishakuyakusan, keishibukuryogan, ninjinto and unkeito on estrogen and progesterone by rat preovulatory follicles. Am J chin med. (1986) 14:161–70. 10.1142/S0192415X86000260 3799534

[41] Usuki S. Effects of hachimijiogan, tokishakuyakusan, keishibukuryogan, ninjinto and unkeito on estrogen and progesterone secretion in preovulatory follicles incubated in vitro. Am J chin med. (1991) 19:65–71. 10.1142/S0192415X91000107 1897493

[42] Usuki S. Effects of tokishakuyakusan and keishibukuryogan on steroidogenesis by rat preovulatory follicles *in vivo*. Am J Chin med. (1990) 18:149–56. 10.1142/S0192415X90000198 2270849

[43] Sakamoto S Kudo H Kawasaki T Kuwa K Kasahara N Sassa S. Effect of a Chinese herbal medicine, Keishi-bukuryo-gan, on the gonadal systems of rats. J Ethnopharmacol. (1988) 23:151–8. 10.1016/0378-8741(88)90002-5 3143030

[44] Wang Z Kanda S Shimono T Dambajamts EU Nishiyama T. The *in vitro* estrogenic activity of the crude drugs found in Japanese herbal medicines prescribed for menopausal syndrome was enhanced by combining them. BMC Complement Altern Med. (2018) 23:107. 10.1186/s12906-018-2170-4 29566679 PMC5865359

[45] Sharma S Mahajan A Tandon VR. Calcitonin gene-related peptide and menopause. J Midlife Health. (2010) 1:5–8. 10.4103/0976-7800.66985 21799630 PMC3139267

[46] Ghatta S Nimmagadda D. Calcitonin gene-related peptide: Understanding its role. Indian J Pharmacol. (2004) 36:277–83.

[47] Hay DL Poyner DR. Calcitonin gene-related peptide, adrenomedullin and flushing. Maturitas. (2009) 64:104–8. 10.1016/j.maturitas.2009.08.011 19762180

[48] Chen JT Shiraki M. Menopausal hot flash and calcitonin gene-related peptide; effect of Keishi-bukuryo-gan, a kampo medicine, related to plasma calcitonin gene related peptide level. Clinical Trian. (2003) 45:199–204. 10.1016/S0378-5122(03)00128-2 12818465

[49] Noguchi M Ikarashi Y Yuzurihara M Kase Y Chen JT Takeda S. Effects of the Japanese herbal medicine Keishi-bukuryo-gan and 17beta-estradiol on calcitonin gene related peptide-induced elevation of skin temperature in ovariectomized rats. J Endoctinol. (2003) 176:359–66. 10.1677/joe.0.1760359 12630921

[50] Noguchi M Ikarashi Y Yuzurihara M Kase Y Takeda S Aburada M. Effects of 17 beta-estradiol and the Japanese herbal medicine Keishi-bukuryo-gan on the release and synthesis of calcitonin gene-related peptide in ovariectomized rats. J Pharmacol Sci. (2003) 93:80–6. 10.1254/jphs.93.80 14501156

[51] Hopkins L. Network pharmacology. Nature Biotechnology. (2007) 25:10. 10.1038/nbt1007-1110 17921993

[52] Roth L Sheffer DJ Kroeze WK. Magic shotguns versus magic bullets: selectively non-selective drugs for mood disorders and schizophrenia. Nature Reviews Drug Discovery. (2004) 3:353–9. 10.1038/nrd1346 15060530

[53] Hopkins L. Network pharmacology: the next paradigm in drug discovery. Nature Chemical Biology. (2008) 4:682–690. 10.1038/nchembio.118 18936753

[54] Luo T Lu Y Yan SK Xiao X Rong XL Guo J. Network Pharmacology in Research of Chinese Medicine Formula: Methodology, Application and Prospective. Chin J Integr Med. (2020) 261:72–80. 10.1007/s11655-019-3064-0 30941682

[55] Zhang G Li Q Chen Q Su SB. Network pharmacology: a new approach for chinese herbal medicine research. Evid Based Complement Alternat Med. (2013) 621423. 10.1155/2013/621423 23762149 PMC3671675

[56] Wang X Shi Y Xu L Wang Z Wang Y Shi W. Traditional Chinese medicine prescription Guizhi Fuling Pills in the treatment of endometriosis. Int J Med Sci. (2021) 18:2401–8. 10.7150/ijms.55789 33967618 PMC8100639

[57] Sugimine R Kikukawa Y Kurihara D Arita R Takayama S Kikuchi A. Kampo medicine prescriptions for hospitalized patients in Tohoku University Hospital. Traditional & Kampo Medicine. (2021). 10.1002/tkm2.129334017789

[58] Nagata Y Goto H Hikiami H Nogami T Fujimoto M Shibahara N. Effect of keishibukuryogan on endothelial function in patients with at least one component of the diagnostic criteria for metabolic syndrome: a controlled clinical trial with crossover design. Evid Based Complement Alternat Med. (2012) 2012:359282. 10.1155/2012/359282 22675380 PMC3364603

[59] Hayashi S Shibutani S Okubo H Shimogawara T Ichinose T Ito Y. Examination of clinical efficacy of keishibukuryogan on non-specific complaints associated with varicose veins of the lower extremity. Ann Vasc Dis. (2014) 7:266–73. 10.3400/avd.oa.14-00055 25298828 PMC4180688

[60] Nakae H Okuyama M Igarashi T. Traumatic lateral abdominal wall hematoma treated with Kampo medicines. Traditional & Kampo Medicine. (2015) 2:102–4. 10.1002/tkm2.102225855820

[61] Kumanomido J Ohe M Shibata R Hattori Y Ishizaki Y Ito S. Effective use of Keishibukuryogan in subcutaneous hematoma after implantable cardiac device surgery in two cases. Intern Med. (2021) 60:755–9. 10.2169/internalmedicine.5677-20 33028772 PMC7990628

[62] Takayama S Tomita N Arita R Ono R Kikuchi A Ishii T. Kampo medicine for various aging-related symptoms: a review of geriatric syndrome. Front Nutr. (2020) 7:86. 10.3389/fnut.2020.00086 32766269 PMC7381143

[63] Nozaki K Hikiami H Goto H Nakagawa T Shibahara N Shimada Y. Keishibukuryogan (gui-zhi-fu-ling-wan), a Kampo formula, decreases disease activity and soluble vascular adhesion molecule-1 in patients with rheumatoid arthritis. Evid Based Complement Alternat Med. (2006) 3:359–64. 10.1093/ecam/nel025 16951720 PMC1513153

[64] Mizawa M Makino T Hikiami H Shimada Y Shimizu T. Effectiveness of keishibukuryogan on chronic-stage lichenification associated with atopic dermatitis. ISRN Dermatol. (2012). 10.5402/2012/158598 23213558 PMC3504367

[65] Fujita K Yamamoto T Kamezaki T Matsumura A. Efficacy of keishibukuryogan, a traditional Japanese herbal medicine, in treating cold sensation and numbness after stroke: clinical improvement and skin temperature normalization in 22 stroke patients. Neurol Med Chir. (2010) 50:1–5. 10.2176/nmc.50.1 20098017

[66] Cho K Kim Y Jung W Kim T. Effect of Gui-zhi-fu-ling-wan on hot flashes in young patients: a retrospective case series. J Acupunct Meridian Stud. (2011) 4:129–33. 10.1016/S2005-2901(11)60019-8 21704956

[67] Ushiroyama T Ikeda A Sakuma K Ueki M. Comparing the effects of estrogen and an herbal medicine on peripheral blood flow in post-menopausal women with hot flashes: hormone replacement therapy and gui-zhi-fu-ling-wan, a Kampo medicine. Am J Chin Med. (2005) 33:259–67. 10.1142/S0192415X05002813 15974485

[68] Shigehara K Izumi K Nakashima K Kawaguchi S Nohara T Kadono Y. Efficacy and safety of keishibukuryogan, a traditional Japanese Kampo medicine, for hot flashes in prostate cancer patients receiving androgen deprivation therapy. Transl Androl Urol. (2020) 9:2533–40. 10.21037/tau-20-901 33457227 PMC7807326

[69] Plotnikoff GA Watanabe K Torkelson C Valleur JL Radosevich DM. The TU-025 keishibukuryogan clinical trial for hot flash management in postmenopausal women: results and lessons for future research. Menopause. (2011) 18:886–92. 10.1097/gme.0b013e31821643d9 21738077 PMC3181094

[70] Saruwatari J Takaishi C Yoshida K Takashima A Fujimura Y Umemoto Y. A herbal-drug interaction study of keishi-bukuryo-gan, a traditional herbal preparation used for menopausal symptoms, in healthy female volunteers. J Pharm Pharmacol. (2012) 64:670–6. 10.1111/j.2042-7158.2011.01443.x 22471362

[71] Yasui T Matsui S Yamamoto S Uemura H Tsuchiya N Noguchi M. Effects of Japanese traditional medicines on circulating cytokine levels in women with hot flashes. Menopause. (2011) 18:85–92. 10.1097/gme.0b013e3181e5063c 20647958

[72] Sun X Li Y Su Q Tian S Cao L. Effects of Guizhifuling pill combined with danazol and gestrinone capsule on immune inflammatory response and angiogenesis in patients with endometriosis. Hebei Med J. (2019) 41:1788–92.

[73] Tao R Yu C. Effects of Guizhi Fuling Pill on MEK-2,p-ERK and VEGF Expression in Patients with Endometriosis. Journal of Liaoning College of Traditional Chinese Medicine. (2016) 18:131–4.

[74] Chang Z. Effect of cassia Twigi Fuling pill on serum leptin, vascular endothelial growth factor, interleukin-8 and ovarian function in patients with endometriosis. Modern Journal of Integrated Traditional Chinese and Western Medicine. (2016) 25:3915–7.

[75] Zhang Y Zhao F Liu W Liu J Fan H. Clinical Efficacy of Mifepristone Combined with Guizhi Fuling Capsule in The Treatment of Endometriosis. Progress in Modern Biomedicine. (2017) 19:3703–6.

[76] Qian J. Clinical study on adding flavor of cassia twigi tuckaing pills in the treatment of endometriosis. Liaoning Journal of Traditional Chinese Medicine. (2000) 4:170.

[77] Terauchi M Hiramitsu S Akiyoshi M Owa Y Kato K Obayashi S. Effects of three Kampo formulae: Tokishakuyakusan (TJ-23), Kamishoyosan (TJ-24), and Keishibukuryogan (TJ-25) on Japanese peri- and postmenopausal women with sleep disturbances. Arch Gynecol Obstet. (2011) 284:913–21. 10.1007/s00404-010-1779-4 21120510

[78] Sakamoto S Yoshino H Shirahata Y Shimodairo K Okamoto R. Pharmacotherapeutic effects of kuei-chih-fu-ling-wan (keishi-bukuryo-gan) on human uterine myomas. Am J Chin Med. (1992) 20:313–7. 10.1142/S0192415X92000333 1471615

[79] Arita R Numata T Saito N Takayama S Togashi T Kaneko S. Development of a medical education program with abdominal palpation simulators to support the understanding of traditional Japanese (kampo) medicine in beginners. Traditional & Kampo Medicine. (2019) 6:1–8. 10.1002/tkm2.123025855820

[80] Yakubo S Ueda Y Muroga K Tanekura N Okudaira T Sasanuma T. Modification of an abdominal diagnosis teaching simulator to reproduce patterns of resistance to pressure. Traditional & Kampo Medicine. (2015) 2:31–4. 10.1002/tkm2.101525855820

